# Phantom and in vivo validation of a novel contactless registration method for robot-assisted SEEG surgery

**DOI:** 10.1186/s41016-025-00401-x

**Published:** 2025-09-16

**Authors:** Feng Hu, Xinwei Li, Shiqiang Wu, Wei Jiang, Kai Shu, Ting Lei

**Affiliations:** 1 Department of Neurosurgery, Tongji Hospital, Tongji Medical College, Huazhong University of Science and Technology, Wuhan, China; 2 Wuhan United Imaging Surgical Co., Ltd., Wuhan, China

**Keywords:** Contactless optical registration, Frameless robotics, Stereo-electroencephalography (SEEG), Registration time, Surgical procedure time, Learning curve

## Abstract

**Background:**

In recent years, robot-assisted stereotactic system has been widely used in stereo-electroencephalogram (SEEG) surgery. However, current registration mainly based on bone-fiducial registration (CBR) is limited by the inconvenient of repeated contact during the process. Here, a novel contactless optical-tracking registration (OTR) were applied for SEEG using robot-assisted stereotactic system. To demonstrate the accuracy and convenience of the novel OTR method compared with contact CBR method during the robot-assisted SEEG in phantom and animal model study.

**Methods:**

A head phantom and 12 Bama pigs (*Sus scrofa domestica*) with six under CBR and six under OTR were selected for the SEEG. Procedures were performed using the robot-assisted stereotactic system in different registration methods. The positioning error and time consumption during the registration process were assessed to compare the accuracy and convenience of OTR and CBR. Besides, two new users for the robot-assisted stereotactic system were selected for the learning curve analysis.

**Results:**

The mean positioning errors in OTR group of the target and entry points were 1.68 ± 0.80 mm and 0.76 ± 0.39 mm. And in CBR group, mean positioning errors of the target and entry points were 1.49 ± 0.79 mm and 0.70 ± 0.33 mm. The registration time of OTR method (99.71 ± 1.08 s) was significantly shorter than that using CBR method (241.29 ± 28.95 s) (*p* value < 0.001). During the learning curve analysis, it is earlier for the users to go under OTR than CBR to reach a preferable entry error of 0.5 mm.

**Conclusion:**

The contactless OTR method can effectively reduce the time consumption during the registration process while maintaining the accuracy with CBR method. The novel method not only simplify the procedure by optical-tracking but also shorten the new user’s learning curve compared with current method.

**Supplementary Information:**

The online version contains supplementary material available at 10.1186/s41016-025-00401-x.

## Background

Epilepsy is one of the most common serious brain disorders with a complex pathogenesis influenced by multiple risk factors and a strong genetic predisposition [1]. About 30% of patients with refractory focal epilepsy required surgical treatment [2, 3]. Pre-surgical evaluation aims to identify the epileptogenic zone and to prevent postoperative neurological deficits [4]. When noninvasive assessments could not identify the epileptogenic region, stereo-electroencephalogram (SEEG) is used as the primary epileptic focal localization technique in many epilepsy centers [5]. It is an interventional invasive evaluation method by placing electrodes into the brain parenchyma [6]. Basically, SEEG inserting electrodes through bolts anchored in the skull [7]. Prior to electrode insertion, matching registration of the patient to the stereotactic robot must be completed, and stereotactic surgery uses a three-dimensional coordinate system to locate targets in the brain and perform operations such as biopsy or implantation of deep brain stimulation electrodes [8]. There has been a recent interest in the application of stereotactic neurosurgical robotic by offering simplified perioperative workflow, shortening operative time, reducing invasiveness, and improving patient comfort compared to traditional frame registration [9, 10].

Nowadays, the accuracy of neurosurgical robotics applications is not necessarily weaker than frame-based stereotactic approaches [11]. Therefore, the focus of the discussion has now shifted to the time efficiency of robotic systems [12–14]. The registration methods between the system and the patient can be divided into contact and contactless registration [15, 16]. Contact registration method based on bone-fiducial so widespread today was face the challenge of complex operational processes and certain technical requirements for the operator [17]. Based on the properties of the contact track of optical method, there have been reported about optical registration method for robot-assisted to realize the “touch-free” registration [18]. If a registration method can simplify the procedure, take less time, and shorten the learning curve of the operator while maintaining accuracy, it will be of great practical importance for the clinical treatment process. In this study, a stereotactic neurosurgery-assisted robot was introduced to perform SEEG acquisition to compare the accuracy and convenience of contact bone-fiducial registration (CBR) and contactless optical-tracking registration (OTR) in phantom and Bama pig model.

## Methods

### System constitution and contactless registration principle

The robot-assisted stereotactic system consists of four main components: (a) a robotic arm, (b) an optical tracking system, (c) a touch screen application for information display and software control, and (d) an operator’s console. The optical tracking system (Polaris Vega XT, Northern Digital Inc.) operates at a sampling frequency of 400 Hz. It has a pyramidal-shaped workspace and a spatial resolution of 0.12 mm. When the camera is 0.95 m (minimum distance) away from the target, the planar monitoring range is 0.48 m × 0.48 m. When the camera is 2.4 m (maximum distance) away from the target, the planar monitoring range is 1.56 m × 1.31 m. The registration algorithm uses the iterative closest point (ICP) method to match the 3D coordinates of retro-reflective markers. Temporal synchronization between the optical tracker and robotic arm is achieved via a hardware clock to ensure < 3 ms latency. Preoperative calibration uses a 12-marker reference phantom (accuracy ± 0.12 mm), with intraoperative recalibration every 30 min via automated marker detection. Error compensation integrates optical tracking data with robotic encoder feedback through extended Kalman filtering, correcting for trajectory deviations in real time.

**Fig. 1:**
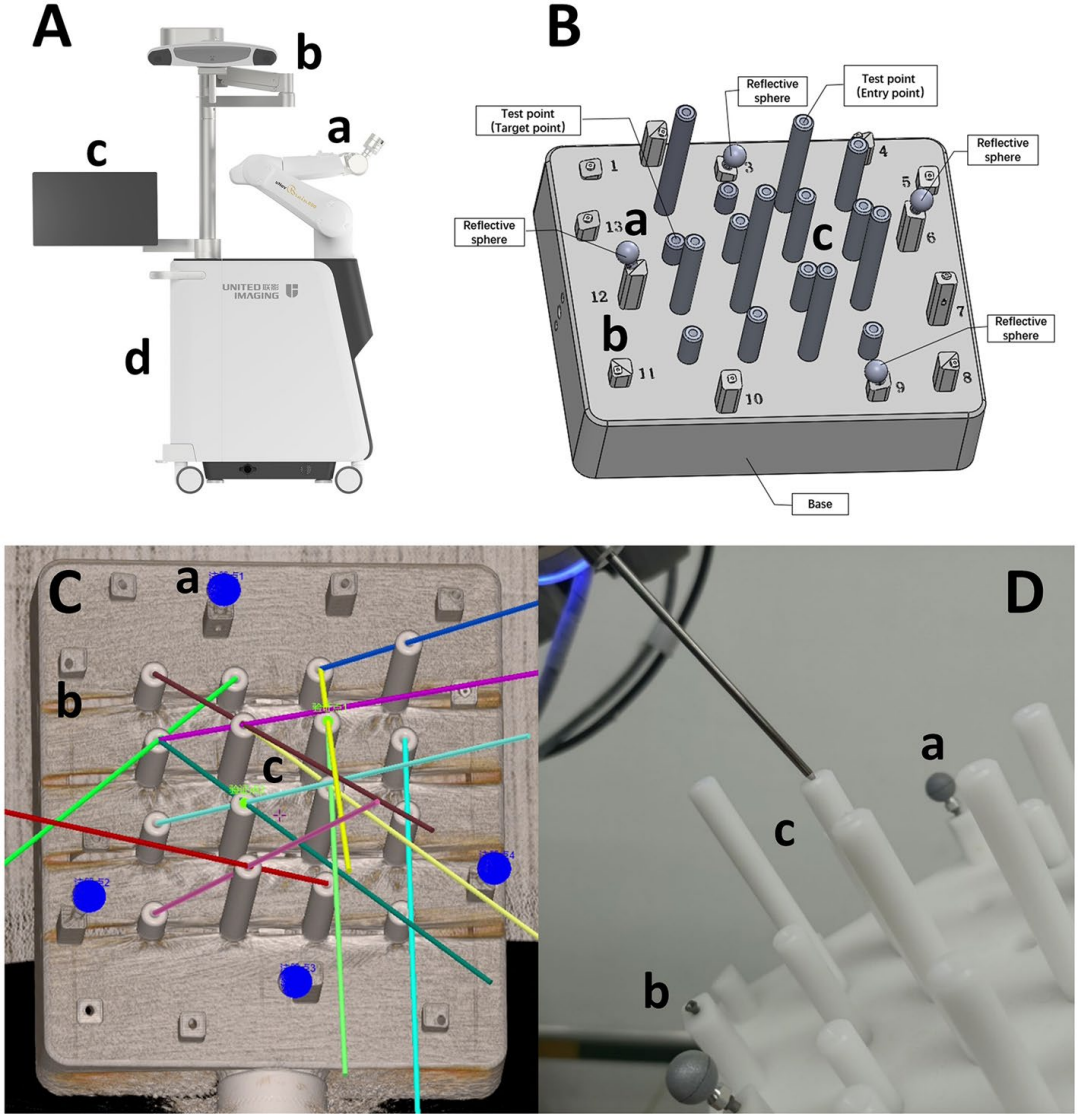
System constitution and phantom verification. **A** Overall view of the robot system: (a) a robotic arm, (b) an optical tracking system, (c) a touch screen application for information display and software control, and (d) an operator’s console. **B** Phantom designed for verification. **C** CT image of phantom for planning trajectories. **D** An electrode guided to the center of phantom verification marker

The whole components of robot-assisted stereotactic system are connected to the cart (Fig. 1A). Beside this, a special array module comprised of several calibration marker spheres with retro-reflective surface was designed and mounted at the (a) 6-degree-of-freedom robotic arm. These calibration marker spheres were used to real-time receive and reflect IR (infrared) light back to the (b) optical tracking system. As a result, the optical tracking system realizes the motion capture of the robotic arm by tracking the light intersections. Specially, several additional calibration marker spheres were prepared and attached to the skull of surgery patients. Due to the same retro-reflective surface, these marker spheres were also tracked by the (b) optical tracking system. Therefore, they were conveniently applied to achieve OTR by of the robot-assisted stereotactic system after the temporal synchronization and accurate spatial calibration during the system development.

### Phantom experiment

A phantom was developed to verify the novel contactless registration method. As shown in Fig. 1B, the phantom was comprised of three components: (a) 4 calibration marker spheres (shown as reflective spheres located at marker No. 3, 6, 9, and 12) as optical fiducial markers for OTR; (b) 9 bone screw markers distributed at other marker points were utilized for CBR; and (c) a batch of different height columns with holes in the range 1–4 mm diameters on the top. These holes were set as test points (including entry points and target points) during the experiment to test the positioning accuracy. These components are all connected to a stable base designed to fix firmly in the robot-assisted stereotactic system to reduced movement errors. During the phantom experiments, two different test points were set as entry and target points respectively each test, and probes with different diameters matching test point holes were selected at the same time. To verify the positioning accuracy of CBR and OTR, defining the target errors as the distance between the center of the holes and the tips of the probes.

### Animal experiment

All of the experimental procedures were complied with Institutional Animal Care and Use Committee (IACUC) of Tongji Medical College, Huazhong University of Science and Technology. Twelve 9-to-13-month-old male Bama pigs (Hubei Yizhicheng Biological Technology Co., Ltd., Hubei, China) with weight ranging from 25 to 35 kg were used in the experiment. After arrival, all the pigs were housed in temperature- and humidity-controlled room (20–25 °C, 50–70% humidity, and 12-h light/12-h dark cycle) with a 7-day previous acclimation period. Pigs were randomly divided into CBR group and OTR group (*n* = 6/group). Before surgery, pigs were fasted for 6–8 h and general anesthetized with intramuscular injection of xylazine (1.5 mg/kg) and Zoletil 50 (zolazepam + tiletamine) (5 mg/kg) for induction. After endotracheal intubation, anesthesia was maintained with 1.5–3% isoflurane in oxygen using an anesthesia machine (A5 Anesthesia System, Mindray Bio-Medical Electronics Co., Ltd., Shenzhen, China). During surgery, the blood oxygen saturation, heart rate, temperature, and respiration rate were continuously monitored by veterinarian staff (iPM 10 Patient Monitor, Mindray Bio-Medical Electronics Co., Ltd., Shenzhen, China). The pigs were monitored after the surgery until they recovered from general anesthesia. Clinical observations of their food intake and behavior were also undertaken for an additional 7-day period. After observations, all the animals were anesthetized by intramuscular injections of ketamine and inhalation of isoflurane and sacrificed by intravenous injection of potassium chloride.

### Preoperative MRI/CT acquisition and surgical planning

Preoperative MRI images were performed on a 3.0-T MRI scanner (Magnetom Skyra, Siemens Healthineers, Erlangen, Germany) with a 32-channel head coil. T1-weighted image was performed with the following parameters: TR/TE = 2300/2.41 ms, slice thickness = 0.80 mm. The parameters for 3D time-of-flight (TOF) magnetic resonance angiography (MRA) were TR/TE = 21/3.69 ms, slice thickness = 0.50 mm; the parameters for Phase Contrast (PC3D) MRV were as follows: TR/TE = 3.4/1.27 ms, slice thickness = 1.10 mm. Detailed description provided in Supplementary information. Preoperative CT acquisition were performed on a CT scanner (SOMATOM Siemens Healthineers, Erlangen, Germany). During the scanning procedure, the Bama pigs were placed on the CT/MRI table in the prone position.

The preoperative MRI and CT images were automatically fused for planning utilizing a stereotactic image-guided surgical planning software (uPlan-Brain prototype; Wuhan United Imaging Surgical Co., Ltd., Wuhan, China). The details of error verification in CT/MRI image fusion were described in Supplementary information (Fig. S1). Additional manual fine-tuning of fusion images was done whenever necessary. Surgical planning was carried out in the robot-assisted stereotactic system (uNav-Brain prototype, Wuhan United Imaging Surgical Co., Ltd., Wuhan, China) based on preoperative MRI/CT fusion images. Based on this system, plans for trajectories of the SEEG electrodes, including the sites of distal target point and cortical entry point, were designed by using image fusion-guided planning sub-software. With the assistance of sub-software, the trajectory of the SEEG electrode was planned to avoid sulci, blood vessels, and screws in fusion images, and the insertion angle was set as close to vertical as possible in order to avoiding tip-shifting during the drilling in high angle insertion. On the other hand, the small angle between the planning trajectory and the skull surface should be avoid either to reduce inconvenience in SEEG electrode implanted After trajectories and coordinates has been created, the sub-software allow surgeons to undertaking a double check and adjustment.

### Registration

To minimize the movement and facilitate the operation during the animal experiment, a head holder was designed to rigidly fix the Bama pig’s head. As shown in Fig. S2, after fixed with the Bama pig’s head, the head holder was also connected firmly with the robot-assisted stereotactic system to further avoid relative movement. Two different registration methods, CBR and the novel contactless OTR, were all performed in phantom and animal experiments respectively to realize comparative experiments. During the CBR procedure, after 4–6 bone fiducials were placed in skull and in contact with robotic pointer, the robot-assisted stereotactic system has completed the CBR procedure by using automatic bone registration sub-software. On the other hand, the OTR procedure avoids the requirement of contacting between robotic pointer and skull by using several calibration marker spheres with retro-reflective surface. More specifically, these calibration marker spheres were attached on the skull and tracked by the optical tracking system contactless through the formation of the light intersects. With the accurate calibration of automatic optical registration sub-software, the spatial registration of the robot-assisted stereotactic system has accomplished.

The CBR and OTR methods were repeated until their spatial registration error realized an acceptable limit at 0.5 mm. Moreover, the centers of the bone fiducials were randomly set as the test targets and were located by the robotic system.

### SEEG implantation

Following registration procedure, all phantom and Bama pigs underwent the SEEG electrode implantation. After registration and disinfection, a 15-mm incision was made over the cranial skin to expose the skull for entry point. To restrict the drilling remain within the thickness limit of the skull, the depth was measured on preoperative CT images by length measurements on program and used to designed a stopper installed on drill bit to restrict drilling depth. Then, a 2.5-mm-diameter hole was created with an orthopedic drill by surgeon. A 1-mm-diameter adaptor was designed to guide the needle probe and adapted with the robot-assisted stereotactic system. Under the guidance of the robotic system, the needle probe was inserted to open the dura mater, and the guide bolt was screwed into the skull. With the guidance of the implanted bolt, a stylet was inserted to generate the initial trajectory. Finally, the SEEG electrode (Rishena Technology Development Co. Ltd., Changzhou, Jiangsu, China) was implanted and secured within the guide bolt. The above processes were repeated until all SEEG electrodes are implanted. The exact implantation depth (*D*) of the electrode can be formulated as follows:

$$D={L}_{1}+{L}_{2}$$

where *L*_1_ is defined as the whole length from the entry point on the surface of the adaptor to the target and evaluated by the program of the robot-assisted stereotactic system, and *L*_2_ is defined as the length from the entry point on the surface of the adaptor to the top of the guide bolt measured by a sterilized steel ruler. Once there were head movements obviously observed or the registration matrix regarded inaccurate by surgeon, accuracy verification was taken by driving the robot arm to specific bone fiducials or anatomical landmark. Intraoperative registration was inspected and the re-registration iterated if deemed necessary.

### Postoperative CT acquisition and measurement

After the SEEG surgery, the postoperative CT acquisition was applied immediately. And the images were co-registered and fused with preoperative images to realize the calculation of positioning error in SEEG implantation. The positioning error (*TE*) is defined as the distance between the target position in the surgical plan and the overserved position of the electrode [19] in postoperative CT and formulated as Euclidean distances as follows:

$$TE=\sqrt[2]{{L}_{m}^{2}+{L}_{n}^{2}}$$

As shown in Fig. S3, where the *L*_*m*_ and *L*_*n*_ indicated the distance in different plane. The results were measured by three different radiologists and took the average of their observation. At the same time, the time consumption was also recorded during the SEEG implanted especially in the process of different registration methods.

The positioning error and time consumption of the OTR method were compared with from CBR method during both phantom and animal experiments. Specially, to verify the rate of progress in gaining experience of the novel contactless registration method, the learning curve of OTR and CBR were plotted and compared by introducing two new users for experiment respectively.

### Statistical analysis

All data were presented as mean ± standard deviation (SD) and analyzed by one-way ANOVA, independent-samples *t*-test, and linear regression. Statistical data analyses were performed using the SPSS 21.0 software (IBM, Chicago, IL, USA). A significant difference was confirmed between groups when *p* < 0.05. All data were plotted using the MATLAB (MathWorks Inc., Natick, MA, USA) and GraphPad Prism version 6.00 (GraphPad Software, La Jolla, CA, USA) software packages.

## Results

### Phantom experiment

**Fig. 2:**
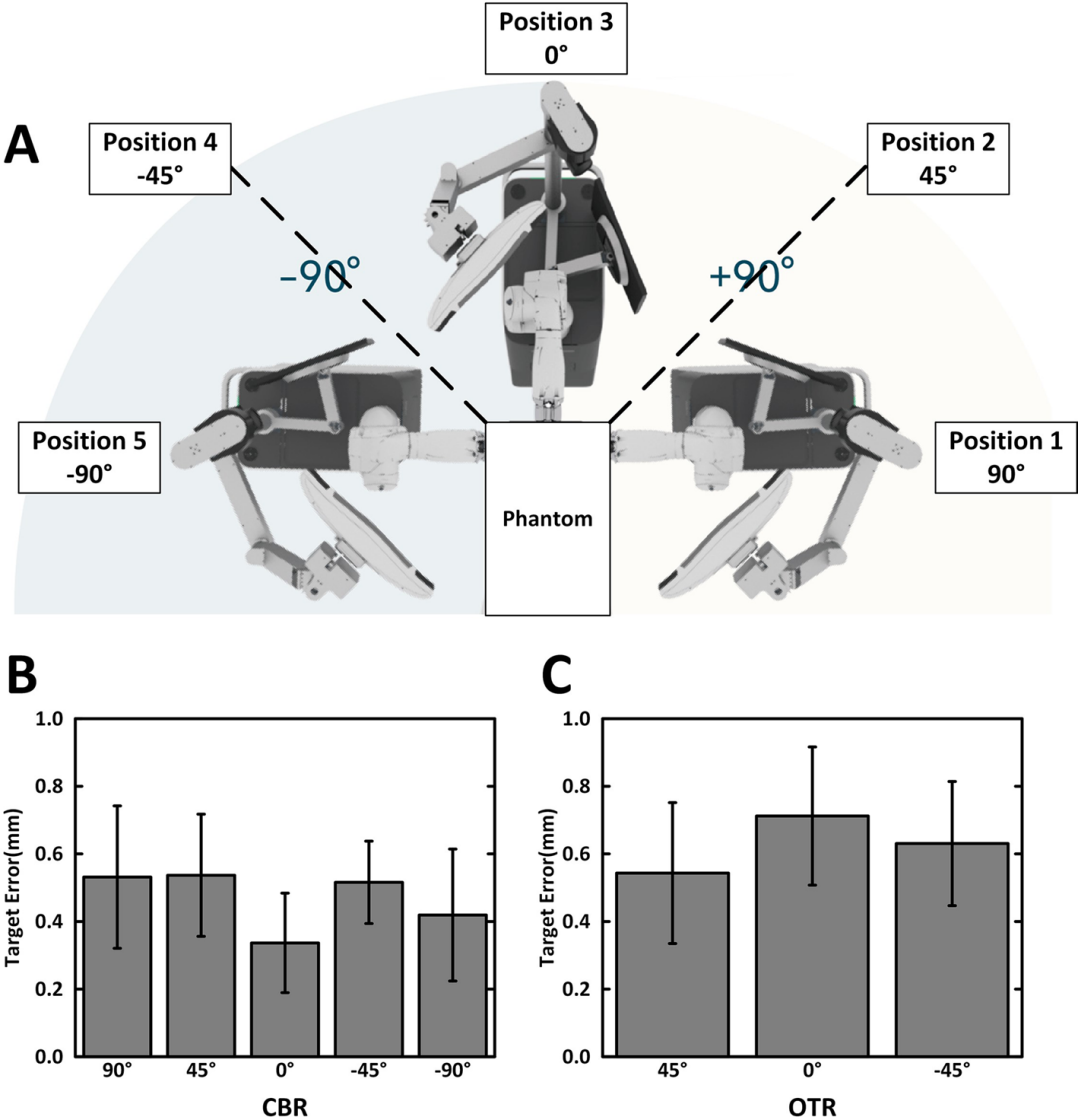
**A** The relative placement between the robot and phantom. Five positions (90°, 45°, 0°, − 45°, − 90°) were selected under CBR and 3 positions (45°, 0°, − 45°) were selected under OTR. **B** Positioning errors of CBR group. **C** Position errors of OTR group

To verify the positioning accuracy in different relative positions between phantom and the robot-assisted stereotactic system, five different system positions relative to phantom (Fig. 2A) were designed to test in different registration methods. Among them, 5 positions were utilized for CBR (positions 1–5), and 3 positions at − 45°, 0°, 45° (positions 2–4) were selected for OTR group. The positions 1 and 5 were excluded from OTR group due to the possibility of blocking view of optical tracking system at ± 90° placement to detect the calibration marker spheres. Position error under CBR are demonstrated in Fig. 2B. Target errors of positions 1–5 are respectively 0.53 ± 0.21 mm, 0.54 ± 0.18 mm, 0.34 ± 0.15 mm, 0.52 ± 0.12 mm, and 0.42 ± 0.20 mm. At the same time, position errors under OTR are demonstrated in Fig. 2C. The target errors of positions 2–4 are respectively 0.54 ± 0.21 mm, 0.71 ± 0.20 mm, and 0.63 ± 0.18 mm. These target errors of CBR or OTR displayed good consistency in different positions. And it also should be noticed that the target errors of OTR achieved the similar accuracy as the traditional CBR.

### Animal experiment

**Fig. 3:**
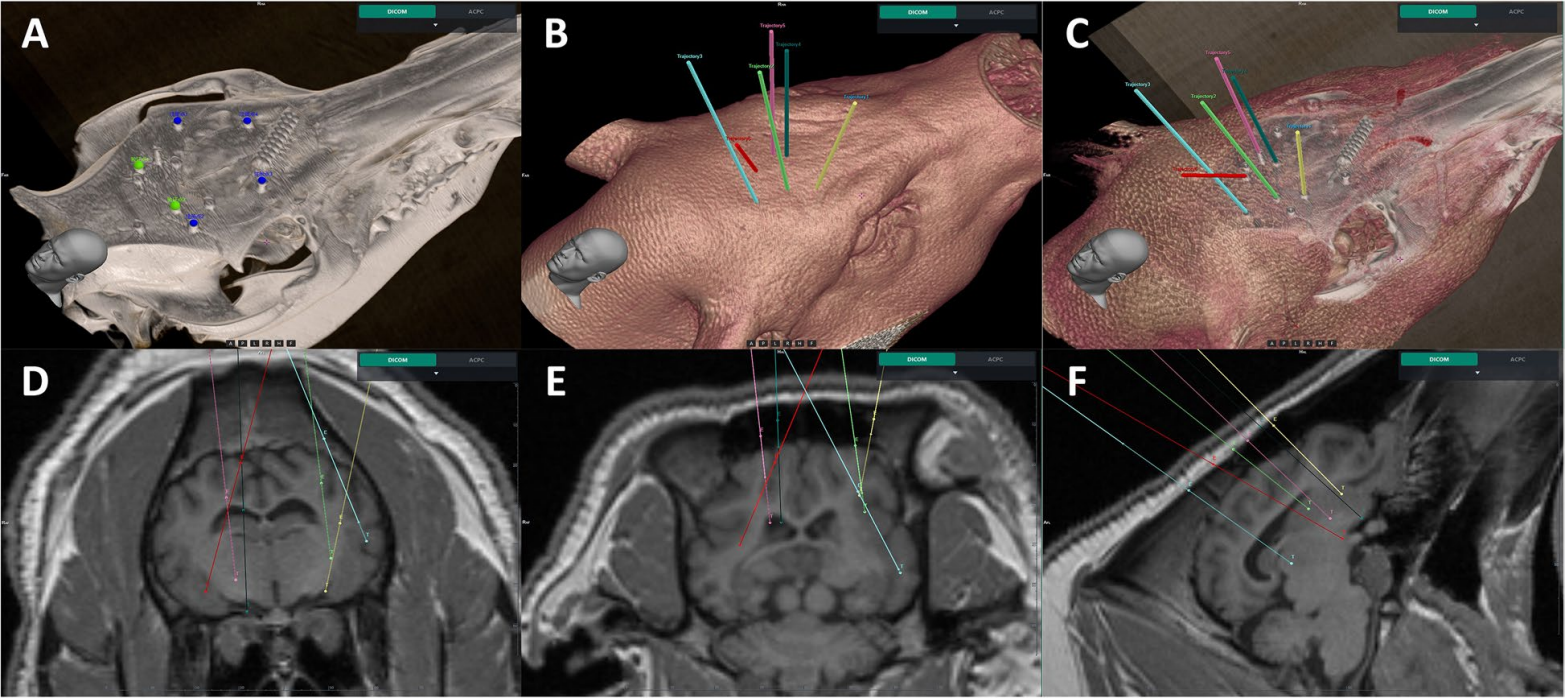
Markers were automatically segmented from preoperative CT image and confirmed by surgeon. **A** Four markers (blue) for registration and two markers (green) for verification. **B** Entry and target points for the electrodes. **C** 3D model reconstructed based on fusion images to check trajectories. **D**, **E**, **F** Transverse section, coronal section, and sagittal section of the electrode placement

The trajectory of the SEEG electrode were planned in the fused images as shown in Fig. 3. The fusion explicitly provided high-quality reference image for subsequent SEEG implantation. At the same time, the SEEG trajectory planning is also clearly visible in the fused image to observing the interrelationship of different trajectories in each electrode.

**Fig. 4:**
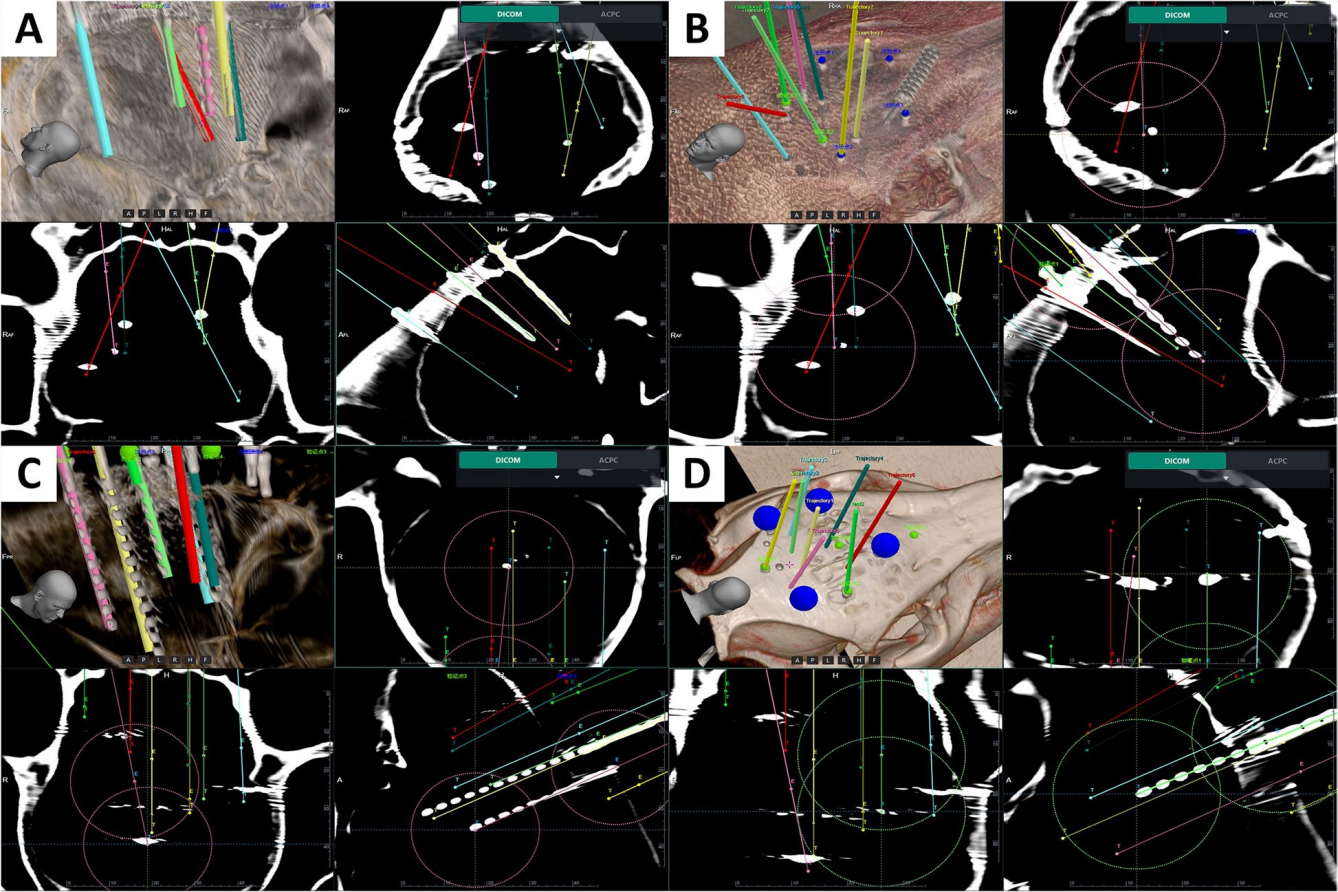
Planning MRI and postoperative CT verification image. **A**, **B** A case of postoperative analysis under CBR. **C**, **D** Another case of postoperative analysis under OTR

After the registration procedure and SEEG implantation, the electrode location were clearly showed in two- and three-dimensional (2D and 3D) views of postoperative CT. The positioning error between the plan and the actual position of the electrode was obtained and recorded for further analysis from postoperative CT and preoperative MR/CT images (Fig. 4).

### Accuracy and time consumption analysis

**Fig. 5:**
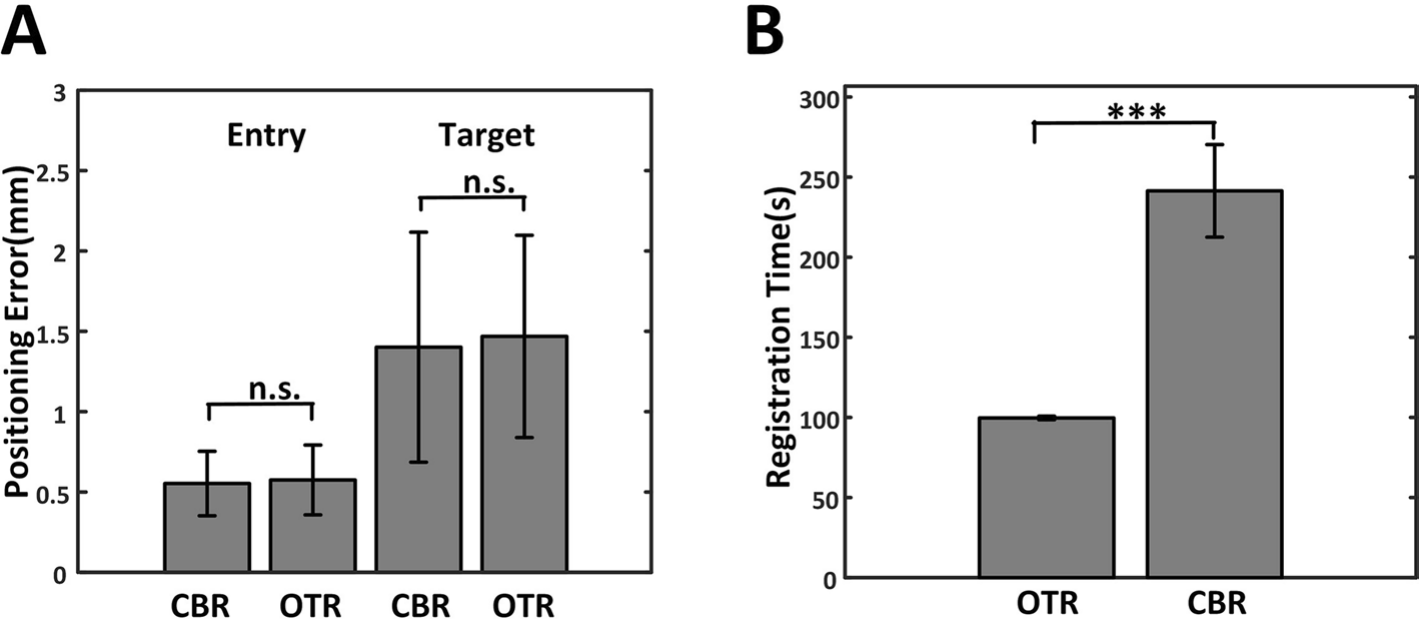
**A** Entry error and target error in the CBR group and the OTR group (n.s. not significant). **B** Registration time consumption comparison (***p value < 0.001)

A total of 72 electrodes were implanted bilaterally in Bama pigs from OTR and CBR groups. The TE of each implantation were recorded and calculated separately. After systematic analysis, the mean TE in OTR group were 1.68 ± 0.80 mm (target point) and 0.76 ± 0.39 mm (entry point) respectively. And in CBR group, mean TE were 1.49 ± 0.79 mm (target point) and 0.70 ± 0.33 mm (entry point). The OTR group exhibits positioning errors values close to the CBR group. No significant differences were observed between the OTR group and the CBR group in either target or entry point errors (*p* values > 0.05) (Fig. 5A).

The registration time of the OTR group and CBR group was also recorded and compared (Fig. 5B). It is noteworthy that the registration time of OTR method (99.71 ± 1.08 s) was significantly shorter than that using CBR method (241.29 ± 28.95 s) (*p* value < 0.001). This is mainly because that registration of OTR relies on optical tacking system which can automatically capture the array of reflective ball makers on the robot.

### Learning curve analysis

**Fig. 6:**
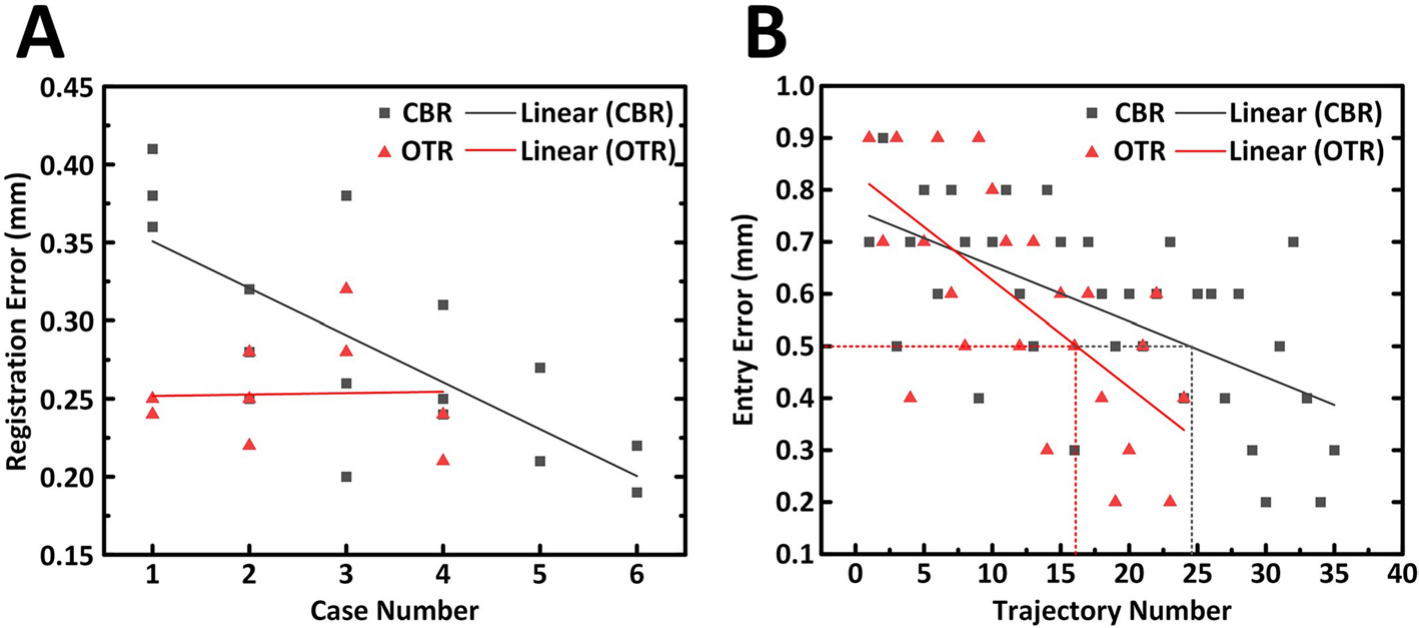
Learning curve analysis of the registration errors and entry errors in CBR and OTR. **A** Linear fitting curves of registration error show their tendency. **B** Entry errors distribution under CBR and OTR. Two solid lines indicates the acceptable entry error defined at 0.5 mm

Two new users of the robot-assisted stereotactic system were selected to complete the SEEG procedure subsequently. The learning curve analysis of CBR and OTR was performed. To analyze the learnability of the different methods, the CBR (16 errors) and OTR (10 errors) registration errors of the new users were recorded and linear fitting in Fig. 6A. As a result, the black solid fitting line indicates that new user need time and cases to practice so to achieved an acceptable margin of errors. On the other hand, linear fitting of the OTR registration error seems to keep constant. Due to the automation of whole OTR process, the new users made registration errors less than 0.3 mm at the first time. As shown in Fig. 6B, the TE of new users in CBR (36 errors) and OTR (24 errors) were also recorded. Entry points are considered better to reflect the system accuracy regardless of the implanting manipulation error. The result showed that the linear fitting of OTR may have a higher slope than CBR which indicated that the OTR can achieved lower errors faster than CBR. Dotted horizontal lines were represented the entry error at 0.5 mm (defined as acceptable errors). Compared to the CBR, the new user in OTR needs less trajectories to realize these acceptable errors. The statistics of regression result in learning curve analysis details is reported in Supplementary information (Fig. S4).

## Discussion

The development of robot-assisted system brought a helpful tool to stereotactic neurosurgery including SEEG [20]. According to previous studies, the robot-assisted system improves patient comfort in surgical process by reducing the time consumed during the surgery process. However, the traditional CBR method needs a process of contact with robotic pointer and leads to inaccuracies occurred during manual trajectory alignment [12, 13]. As a result of relying on automatic optical system, OTR method can effectively reduce the operator dependence and can overcome human physiological fatigue registration. And it may also be applied in future scenarios other than SEEG, such as DBS and brain biopsy, and may also have potential application value in orbital and craniofacial surgery [21]. Therefore, its development has great significance for the promotion of robot-assisted system [22, 23].

In this study, a novel robot system with OTR method for SEEG implantation is reported. In the animal experiment, we used 12 Bama pigs for SEEG surgery by OTR method and CBR method respectively. During the experiment, the accuracy and time consumption of registration has been evaluated and compared. The result indicated that OTR method shows high accuracy with no significant differences observed between the OTR group and the CBR group in either target or entry errors. Nevertheless, OTR method significantly reduces the time consumption of registration process compared with CBR method. Meanwhile, it takes fewer cases to practice for the users to achieve a preferable registration error and entry error. Beyond time efficiency, OTR offers additional advantages: (i) simplified workflow (reducing procedural steps from 12 to 5 compared to CBR), lowering operator fatigue; (ii) minimized risk of iatrogenic infection by eliminating direct contact between the robotic pointer and skull; and (iii) enhanced scalability, as shown by the steeper learning curve for new users (achieving 0.5 mm entry error in 50% fewer cases than CBR). These benefits align with recent trends in robot-assisted neurosurgery emphasizing both precision and workflow optimization [21, 24].

Our results are partially concordant to previous studies. In a phantom study of ROSA stereotactic robot, the mean target accuracy was 1.59 mm for 3 T MRI-guided frameless surgery, 0.3 mm for flat-panel computed tomography (fpCT)-guided frameless surgery, and 0.3 mm for CT-guided frame-based surgery [24]. Another study of ROSA robot reported the time efficiency of robot applications and the essential learning curve of the surgeon with a similar bone fiducial based registration time of 3.5 ± 1.1 min and registration error of 0.42 ± 0.15 mm [25]. In contrast to the contactless laser surface registration (LSR) in ROSA, the proposed OTR method in the article could shorten the time consumption of registration within 2 min (LSR: 16.7 ± 2.3 min) and decrease the registration error below 0.35 mm (LSR: 0.60 ± 0.17 mm). A neurosurgical robot in China has been reported Euclidean errors of target and entry points of 2.68 ± 1.03 mm and 1.47 ± 0.63 mm respectively in hippocampus-SEEG in nonhuman primates [26].

Nevertheless, there are still some limitations in this study. Bama pigs provide a valid preclinical model due to similarities in brain volume and vascular distribution, key differences persist—including cortical thickness, white matter fiber density, and gyral complexity—that may affect translational relevance [27–29]. Subsequent clinical trials may be considered to verify the advantages of the contactless registration method. It can also make more preparations for the preoperative path planning, optimize the image algorithm [30, 31], and improve the actual operation methods during the operation. We are initiating a multicenter clinical trial which involving 50 patients with refractory epilepsy, aiming to validate OTR in human SEEG procedures.

In conclusion, the accuracy of the contactless OTR method in this study can meet the requirements of robot-assisted SEEG surgery. The application of OTR in phantom and animals exhibited accuracy, safety, and effectiveness. Compared with the traditional CBR method, it can greatly simplify the surgical process for clinician during manual trajectory alignment. It can also shorten the learning-time consumption for new users in the registration of robotic directional surgery technology. The novel method may benefit the efficient conversion of allocation of medical resources and the reduction of medical expenses.

## Conclusion

In this study, a novel contactless registration method has been applied for robotic-assisted SEEG in both phantom and animal model. The application of contactless OTR methods presented consistent accuracy and less time consumption compared with the traditional CBR methods. On the other hand, it also presented great learnability for new users of this novel registration method in learning curve analysis. In conclusion, the novel OTR method exhibited the possibility in process simplified, time saved, and learnability. This indicated the promising application prospects of OTR method in robot-assisted SEEG surgery.

## Supplementary Information

Additional file 1: Fig. S1 The error verification of CT and MRI fusion test of planning software. Fig. S2 Cranial stabilization system for the Bama pigs’ head. Fig. S3 The Euclidean distances between target position in the surgical plan and overserved position of the electrode. Fig. S4 The statistics of regression result in learning curve analysis shown in Fig. 6.

## Data Availability

The datasets used and/or analyzed during the current study are available from the corresponding author on reasonable request.

## References

[1] Thijs RD, Surges R, O’Brien TJ, Sander JW. Epilepsy in adults. Lancet. 2019;393(10172):689–701. 30686584 10.1016/S0140-6736(18)32596-0

[2] Harroud A, Bouthillier A, Weil AG. Nguyen DK Temporal lobe epilepsy surgery failures: a review. Epilepsy Res Treat. 2012;2012:201651. 22934162 10.1155/2012/201651 PMC3420575

[3] Wiebe S, Jette N. Pharmacoresistance and the role of surgery in difficult to treat epilepsy. Nat Rev Neurol. 2012;8(12):669–77. 22964510 10.1038/nrneurol.2012.181

[4] Baumgartner C, Koren JP, Britto-Arias M, Zoche L, Pirker S. Presurgical epilepsy evaluation andepilepsy surgery. F1000Res. 2019;8:F1000 Faculty Rev-1818. 10.12688/f1000research.17714.1 PMC6820825 31700611

[5] Kim W, Shen MY, Provenzano FA, Lowenstein DB, McBrian DK, Mandel AM, et al. The role of stereo-electroencephalography to localize the epileptogenic zone in children with nonlesional brain magnetic resonance imaging. Epilepsy Res. 2022;179:106828. 34920378 10.1016/j.eplepsyres.2021.106828

[6] Shah AK, Mittal S. Invasive electroencephalography monitoring: Indications and presurgical planning. Ann Indian Acad Neurol. 2014;17(Suppl 1):S89. 24791095 10.4103/0972-2327.128668 PMC4001224

[7] Vakharia VN, Duncan JS. Automation advances in stereoelectroencephalography planning. Neurosurg Clin N Am. 2020;31(3):407–19. 32475489 10.1016/j.nec.2020.03.005 PMC7902942

[8] Lozano AM, Gildenberg PL, Tasker RR. Textbook of stereotactic and functional neurosurgery. Berlin: Springer; 2009.

[9] Leal Ghezzi T, Campos Corleta O. 30 years of robotic surgery. World J Surg. 2016;40:2550–7. 27177648 10.1007/s00268-016-3543-9

[10] Faria C, Erlhagen W, Rito M, De Momi E, Ferrigno G, Bicho E. Review of robotic technology for stereotactic neurosurgery. IEEE Rev Biomed Eng. 2015;8:125–37. 25955851 10.1109/RBME.2015.2428305

[11] Morgan PS, Carter T, Davis S, Sepehri A, Punt J, Byrne P, et al. The application accuracy of the Pathfinder neurosurgical robot. Int Congress Ser. 2003;1256:561–7.

[12] Smith JA, Jivraj J, Wong R, Yang V. 30 years of neurosurgical robots: review and trends for manipulators and associated navigational systems. Ann Biomed Eng. 2016;44(4):836–46. 26467553 10.1007/s10439-015-1475-4

[13] Bekelis K, Radwan TA, Desai A, Roberts DW. Frameless robotically targeted stereotactic brain biopsy: feasibility, diagnostic yield, and safety. J Neurosurg. 2012;116(5):1002–6. 22404667 10.3171/2012.1.JNS111746

[14] Fiani B, Quadri SA, Farooqui M, Cathel A, Berman B, Noel J, et al. Impact of robot-assisted spine surgery on health care quality and neurosurgical economics: a systemic review. Neurosurg Rev. 2020;43(1):17–25. 29611081 10.1007/s10143-018-0971-z

[15] Meng F, Zhai F, Zeng B, Ding H, Wang G. An automatic markerless registration method for neurosurgical robotics based on an optical camera. Int J Comput Assist Radiol Surg. 2018;13(2):253–65. 29101639 10.1007/s11548-017-1675-5

[16] Brodie J, Eljamel S. Evaluation of a neurosurgical robotic system to make accurate burr holes. Int J Med Robot. 2011;7(1):101–6. 21341368 10.1002/rcs.376

[17] Chen ECS, Ma B, Peters TM. Contact-less stylus for surgical navigation: registration without digitization. Int J Comput Assist Radiol Surg. 2017;12:1231–41. 28386757 10.1007/s11548-017-1576-7

[18] Cardinale F, Rizzi M, d’Orio P, et al. A new tool for touch-free patient registration for robot-assisted intracranial surgery: application accuracy from a phantom study and a retrospective surgical series[J]. Neurosurg Focus. 2017;42(5):E8. 28463615 10.3171/2017.2.FOCUS16539

[19] Zettinig O, Frisch B, Virga S, Esposito M, Rienmüller A, Meyer B, et al. 3D ultrasound registration-based visual servoing for neurosurgical navigation. Int J Comput Assist Radiol Surg. 2017;12(9):1607–19. 28236117 10.1007/s11548-017-1536-2

[20] Guo Z, Leong MC, Su H, Kwok KW, Chan DT, Poon WS. Techniques for stereotactic neurosurgery: beyond the frame, toward the intraoperative magnetic resonance imaging–guided and robot-assisted approaches. World Neurosurg. 2018;116:77–87. 29730102 10.1016/j.wneu.2018.04.155

[21] Schreurs R, Baan F, Klop C, Dubois L, Beenen LFM, Habets PEMH, et al. Virtual splint registration for electromagnetic and optical navigation in orbital and craniofacial surgery. Sci Rep. 2021;11(1):10406. 34001966 10.1038/s41598-021-89897-8 PMC8128880

[22] Zhang D, Li Z, Chen K, Xiong J, Zhang X, Wang L. An optical tracker based robot registration and servoing method for ultrasound guided percutaneous renal access. Biomed Eng Online. 2013;12:47. 23705678 10.1186/1475-925X-12-47 PMC3679870

[23] Carl G, Reitz D, Schönecker S, Pazos M, Freislederer P, Reiner M, et al. Optical surface scanning for patient positioning in radiation therapy: a prospective analysis of 1902 fractions. Technol Cancer Res Treat. 2018;17:1533033818806002. 30453842 10.1177/1533033818806002 PMC6243634

[24] Lefranc M, Capel C, Pruvot AS, Fichten A, Desenclos C, Toussaint P, et al. The impact of the reference imaging modality, registration method and intraoperative flat-panel computed tomography on the accuracy of the ROSA® stereotactic robot. Stereotact Funct Neurosurg. 2014;92(4):242–50. 25170634 10.1159/000362936

[25] Machetanz K, Grimm F, Schuhmann M, Tatagiba M, Gharabaghi A, Naros G. Time efficiency in stereotactic robot-assisted surgery: an appraisal of the surgical procedure and surgeon’s learning curve. Stereotact Funct Neurosurg. 2021;99(1):25–33. 33017833 10.1159/000510107

[26] Zhu GY, Chen YC, Du TT, Liu DF, Zhang X, Liu YY, et al. The accuracy and feasibility of robotic assisted lead implantation in nonhuman primates. Neuromodulation. 2019;22(4):441–50. 31012530 10.1111/ner.12951

[27] Sauleau P, Lapouble E, Val-Laillet D, Malbert CH. The pig model in brain imaging and neurosurgery. Animal. 2009;3(8):1138–51. 22444844 10.1017/S1751731109004649

[28] Van Gompel JJ, Bower MR, Worrell GA, Stead M, Chang SY, Goerss SJ, et al. Increased cortical extracellular adenosine correlates with seizure termination. Epilepsia. 2014;55(2):233–44. 24483230 10.1111/epi.12511 PMC4104491

[29] Raghuram H, Looi T, Pichardo S, Waspe AC, Drake JM. A robotic MR-guided high-intensity focused ultrasound platform for intraventricular hemorrhage: assessment of clot lysis efficacy in a brain phantom. J Neurosurg Pediatr. 2022;30(6):586–94. 36115058 10.3171/2022.8.PEDS22144

[30] Azam MA, Khan KB, Salahuddin S, Rehman E, Khan SA, Khan MA, et al. A review on multimodal medical image fusion: compendious analysis of medical modalities, multimodal databases, fusion techniques and quality metrics. Comput Biol Med. 2022;144:105253. 35245696 10.1016/j.compbiomed.2022.105253

[31] Kavita P, Alli DR, Rao AB. Study of image fusion optimization techniques for medical applications. Int J Cogn Comput Eng. 2022;3:136–43.

