# Supplementary figures and images for: Phantom and in vivo validation of a novel contactless registration method for robot-assisted SEEG surgery
Source: Chin Neurosurg J. 2025 Sep 16;11:20. doi: 10.1186/s41016-025-00401-x (PMC12439361; doi:10.1186/s41016-025-00401-x)

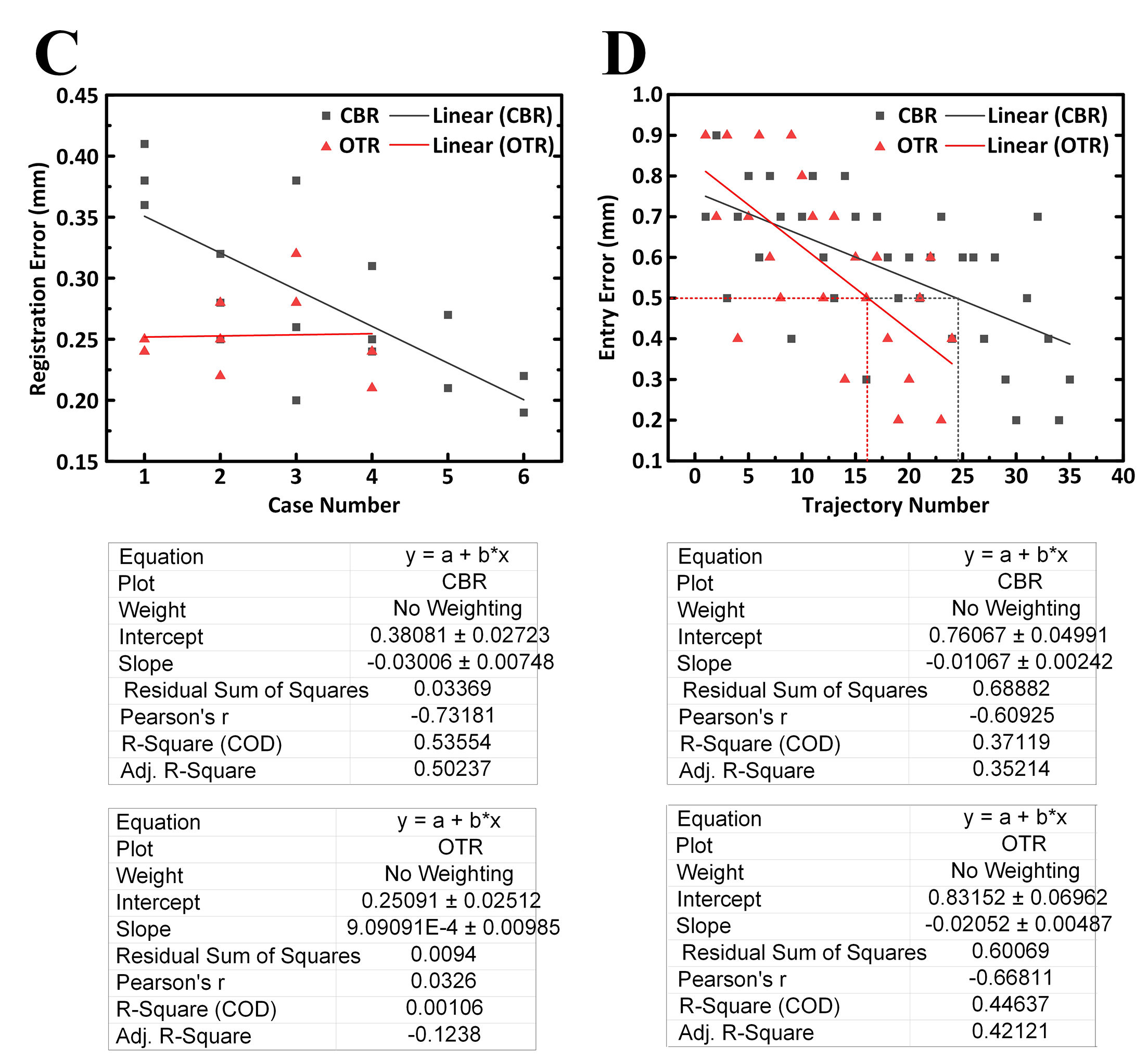

Supplement: Supplementary file 1 — Additional file 1: Fig. S1 The error verification of CT and MRI fusion test of planning software. Fig. S2 Cranial stabilization system for the Bama pigs’ head. Fig. S3 The Euclidean distances between target position in the surgical plan and overserved position of the electrode. Fig. S4 The statistics of regression result in learning curve analysis shown in Fig. 6. [file 41016_2025_401_MOESM1_ESM.zip › FigS1_ESM.tif]

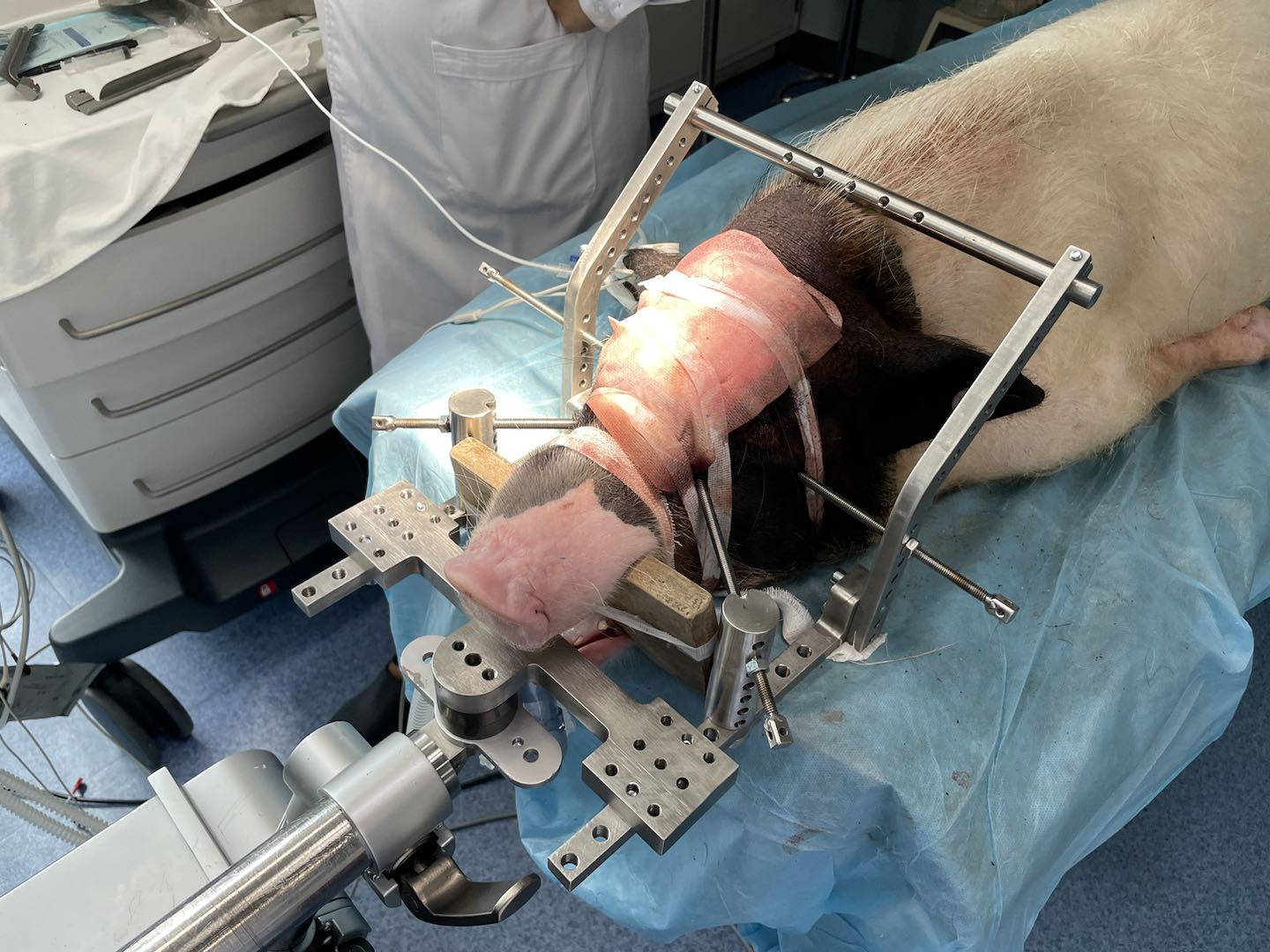

Supplement: Supplementary file 1 — Additional file 1: Fig. S1 The error verification of CT and MRI fusion test of planning software. Fig. S2 Cranial stabilization system for the Bama pigs’ head. Fig. S3 The Euclidean distances between target position in the surgical plan and overserved position of the electrode. Fig. S4 The statistics of regression result in learning curve analysis shown in Fig. 6. [file 41016_2025_401_MOESM1_ESM.zip › FigS2_ESM.tif]

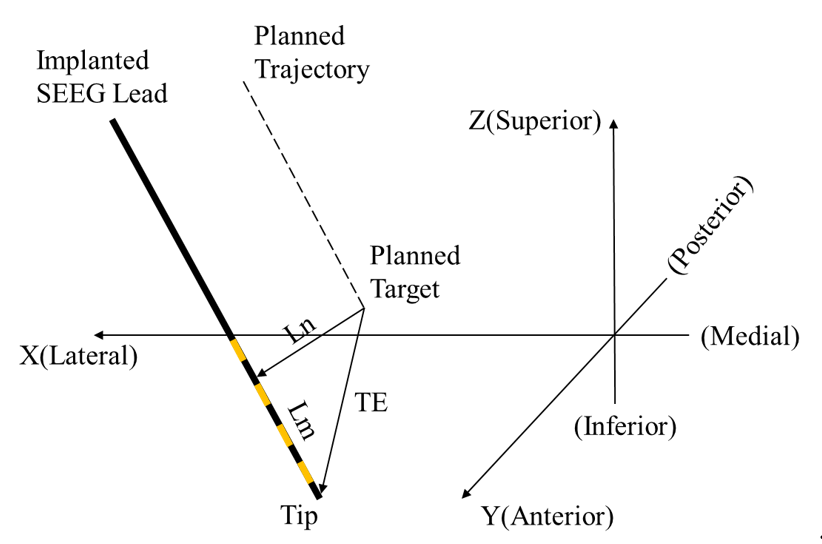

Supplement: Supplementary file 1 — Additional file 1: Fig. S1 The error verification of CT and MRI fusion test of planning software. Fig. S2 Cranial stabilization system for the Bama pigs’ head. Fig. S3 The Euclidean distances between target position in the surgical plan and overserved position of the electrode. Fig. S4 The statistics of regression result in learning curve analysis shown in Fig. 6. [file 41016_2025_401_MOESM1_ESM.zip › FigS3_ESM.tif]

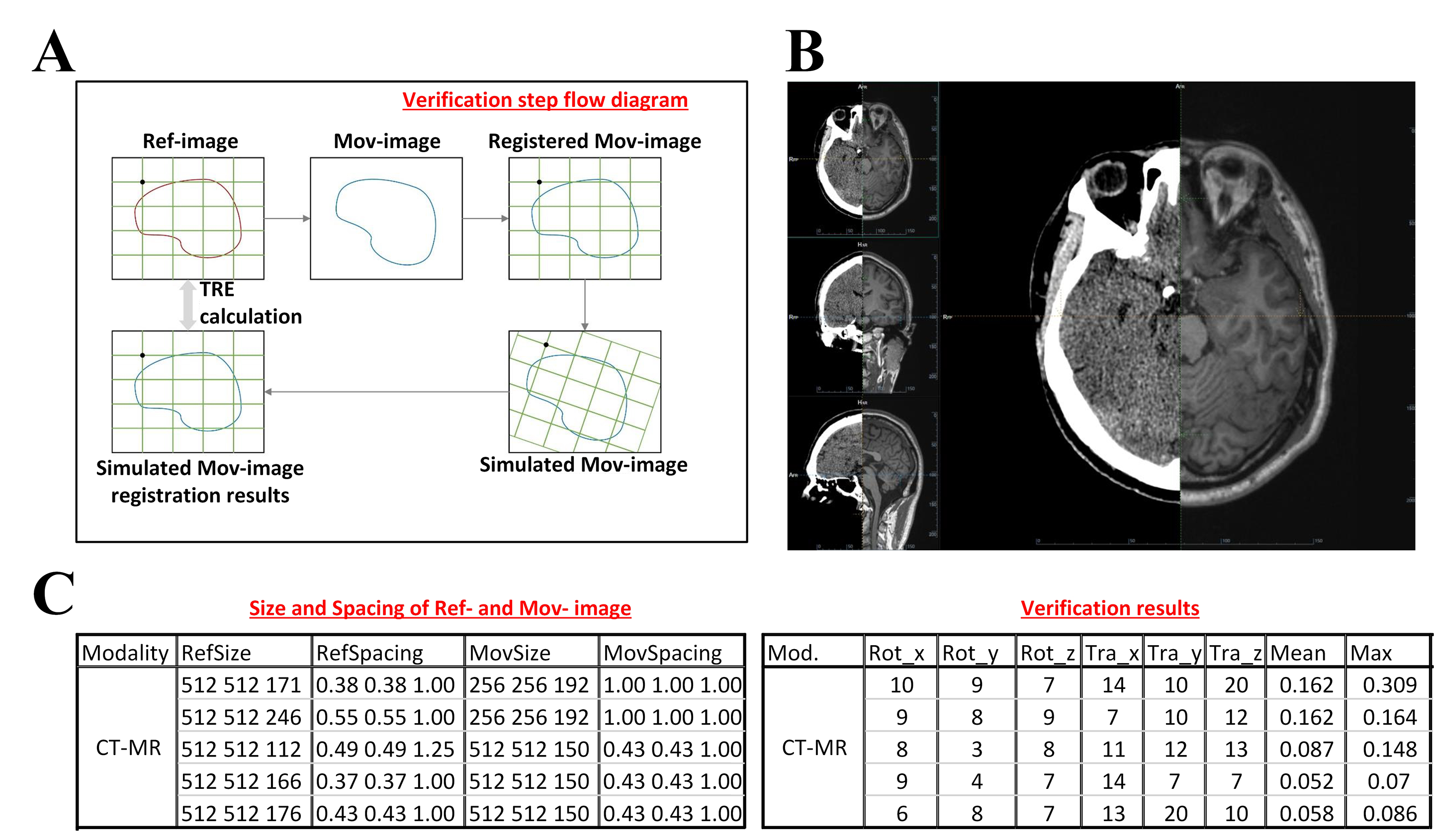

Supplement: Supplementary file 1 — Additional file 1: Fig. S1 The error verification of CT and MRI fusion test of planning software. Fig. S2 Cranial stabilization system for the Bama pigs’ head. Fig. S3 The Euclidean distances between target position in the surgical plan and overserved position of the electrode. Fig. S4 The statistics of regression result in learning curve analysis shown in Fig. 6. [file 41016_2025_401_MOESM1_ESM.zip › FigS4_ESM.tif]
